# What’s what in a pandemic? Virus, disease, and societal disaster must be differentiated

**DOI:** 10.1371/journal.pbio.3002130

**Published:** 2023-05-25

**Authors:** Alexander E. Gorbalenya, Stanley Perlman

**Affiliations:** 1 Department of Medical Microbiology, Leiden University Medical Center, Leiden, the Netherlands; 2 Faculty of Bioengineering & Bioinformatics, Lomonosov Moscow State University, Moscow, Russia; 3 Department of Microbiology and Immunology, Department of Pediatrics, University of Iowa, Iowa City, Iowa, United States of America

## Abstract

Viruses, the diseases they can trigger, and the possible associated societal disaster represent different entities. To engage with the complexities of viral pandemics, we need to recognize each entity by using a distinctive name.

## Abstract 2

Names matter. In a pandemic, the virus, disease, and societal disaster each represent a different entity that should be recognized with distinctive names. This Perspective calls for a rethink of how we use names in a pandemic, using the COVID-19 pandemic as an example.

We live in the virosphere, a world of viruses that depend on hosts for reproduction [1]. They may be invisible to the naked eye, but viruses have shaped the evolution of all life forms and define well-being in many ways that researchers are only starting to appreciate through studies of viromes and hosts [2]. Notwithstanding this recent advancement, viruses are usually perceived as agents of infectious diseases. Infected people may sicken and die, and sharp increases in numbers of diseased and dead globally, compared to the annual average, define pandemics. They create distress leading to adverse societal outcomes that may range from mild to disaster. The latter is most impactful and was evident during the pandemic of severe acute respiratory syndrome coronavirus 2 (SARS-CoV-2) and coronavirus disease 2019 (COVID-19) [3]. We contend that using proper names for the virus, disease, and disaster acknowledges that they are separate entities, representing biological species, organism condition, and societal disruption. This recognition is crucial for untangling and communicating the numerous roles of humans and virus during pandemics, as well as for studying virus–host interactions generally, as we illustrate using the example of the COVID-19 pandemic.

Viruses are the smallest known self-replicating entities that reproduce within cells of host organisms [4]. They were discovered as agents causing infectious diseases that spread through direct or indirect contact in a population. Within this traditional disease-centric framework, a virus is the sole etiology of a disease arising from infection. This link is cemented in the tightly coupled names of a virus and the associated disease. Names typically refer to a disease syndrome or, occasionally, a geographic location or original host where a disease was discovered; for instance, severe acute respiratory syndrome (SARS) and SARS coronavirus (SARS-CoV), and Middle East respiratory syndrome (MERS) and MERS coronavirus (MERS-CoV). Because of the dominant perception of the virus being an attribute of the associated disease and the considerable overlap of their names, the disease names (SARS or MERS in the above examples) are omnipresent in reference to the respective virus.

However, the disease-centric concept is not compatible with recent advances in biomedical science. Disease develops independently in each infected individual, who may also remain healthy [5]. Furthermore, many viruses have been identified that are not known to cause disease, mostly in studies of microbiomes [2]. Thus, the application of disease-centric language to describing viral infections diminishes the complex relationship between viruses and diseases and is misleading. For these reasons, having different names for the disease and for the virus would make it straightforward to differentiate them. A well-known example of this differentiation is acquired immunodeficiency syndrome (AIDS), the disease, and human immunodeficiency virus 1 (HIV-1), its causative agent. Furthermore, naming a virus after its species and not the disease, as is common for nonvirus entities, would facilitate the appreciation of viruses as biological entities. This is the case with SARS-CoV-2 (a virus), which is named after species *Severe acute respiratory syndrome-related coronavirus*, genus *Betacoronavirus*, family *Coronaviridae*. The disease (COVID-19) was given a name that differed from that of the associated virus (SARS-CoV-2), using considerations to avoid stigmatization [6]. For the first time, different names were introduced simultaneously for a virus and a disease. Importantly, SARS-CoV-2 should not be replaced with the term “coronavirus” as this identifies the virus with the family *Coronaviridae*, which includes thousands of viruses; its use is therefore imprecise and may be misleading.

After 3 years of the COVID-19 pandemic, the use of “COVID-19” greatly outnumbers “SARS-CoV-2,” especially at the science–societal interface. While this disparity could reflect reality, we believe that it is largely due to massive amounts of incorrect references to “SARS-CoV-2” as “COVID-19” (Table 1). This conclusion is supported by the striking propagation of “COVID-19 coronavirus” or “COVID-19 virus” in communications. These terms are incorrect and grammatically awkward, but reinforce the traditional link between the virus and the disease, which have made them appealing to use.

**Table 1:** Common inaccurate or ambiguous terms complicating communication about critical aspects of the COVID-19 pandemic.

Domain	Correct term	Inaccurate term	Comment
**Virus**	SARS-CoV-2	COVID-19 coronavirus; COVID-19 virus; Coronavirus^1^; Coronavirus SARS-CoV-2	SARS-CoV-2 term is the acronym of the virus name (see text). SARS-CoV-2 has become one of many human coronaviruses. The COVID-19 term is the acronym of the disease name (see text). There are several naming systems for genetic variants of SARS-CoV-2 but none that are comparable for COVID-19.
	SARS-CoV-2 variants	COVID-19 variants	
	Reproduction rate of SARS-CoV-2	Reproduction rate of COVID-19	
**Disease**	COVID-19	Coronavirus disease; COVID	COVID-19 is triggered by SARS-CoV-2 infection in humans. Clinical manifestation is required to declare a person unwell. Infected individuals may stay healthy (asymptomatic) due to many different and poorly understood factors.
	Asymptomatic (SARS-CoV-2 case)	Asymptomatic (COVID-19 case)	
**Diagnostic**	SARS-CoV-2 test	COVID-19 test; Coronavirus test	Three main molecular diagnostic tools, namely PCR, antigen tests, and antibody tests, recognize either SARS-CoV-2 components or host responses to the virus. They assist with discriminating COVID-19 from other diseases with similar clinical manifestations.
	SARS-CoV-2 positive	COVID-19 positive	
	SARS-CoV-2 infected	COVID-19 infected	
	No longer SARS-CoV-2 positive	Recovered from COVID-19^2^	
**Epidemiology**	SARS-CoV-2 origin	COVID-19 origin	It is the virus rather than the disease that is transmitted between individuals. Due to practical considerations, PCR is the leading test for counting those infected by SARS-CoV-2 (cases).
	SARS-CoV-2 hot spots	COVID hot spots	
	SARS-CoV-2 infection	COVID infection	
	SARS-CoV-2 cases	COVID-19 cases^3^	
	Number of SARS-CoV-2 PCR positives	Case number	
**Vaccine**	SARS-CoV-2 vaccine	COVID-19 vaccine; Coronavirus vaccine	All available vaccines aim to trigger cellular and humoral immune responses against SARS-CoV-2 protein(s). They affect virus reproduction directly and COVID-19 development only indirectly.
	Anti-SARS-CoV-2 antibody	COVID antibody	
**Drugs**	Anti-SARS-CoV-2 drug	COVID-19 drug^4^	Most drugs target SARS-CoV-2 replication and only indirectly affect COVID-19 development.

^1^In communications about SARS-CoV-2 when it is not specified in the first place.

^2^This expression may mean that a patient is free from COVID-19 syndromes, especially in a hospital setting.

^3^This term is accurate to account for sick with COVID-19.

^4^All drugs used to treat COVID-19 were either already in use to control abnormal organismal conditions, such as inflammation or blood coagulation, or were developed to block SARS-CoV-2 reproduction.

This tradition may also explain other numerous misreferences to “COVID-19” when “SARS-CoV-2” is correct and should be used (Table 1). For diagnostic assays, SARS-CoV-2 is the actual target, whereas COVID-19 motivated the assay development. Also, asymptomatic individuals who test positive for SARS-CoV-2 infection are commonly referred to as “sick with COVID-19” or “COVID-19 positive” and counted toward cumulative COVID-19 cases in many tallies, thereby inflating the disease burden; they should instead be designated as “SARS-CoV-2 cases.” This misrepresentation of diagnostic results contradicts the general definition of disease as an abnormal condition and is poorly compatible with the observation that healthy humans carry many viruses over a lifetime. It undermines the importance of separate tallies for symptomatic and asymptomatic individuals, the latter of which are usually undercounted [7].

Likewise, combating COVID-19 motivated research and applications, but it is SARS-CoV-2 that is the target of vaccines and most drugs, which make these controlling measures indirect in respect to the disease (Table 1). In addition to anti-SARS-CoV-2 agents that are directed at SARS-CoV-2 components, so-called “COVID-19 drugs” include others that target either the host response to the virus in a nonspecific manner or a host factor essential for viral reproduction. Some of these are already available (e.g., anticoagulants or anti-inflammatory drugs), and others will be developed in the future. None of these drugs and vaccines are specific for the prevention or treatment of COVID-19 and may misinform when labelled as “COVID-19-directed.” On a general note, labeling vaccines and drugs after their direct target, SARS-CoV-2 or a host factor(s) [8], would promote awareness about the complex virus–disease relationship and facilitate developing realistic expectations and building trust in control measures. Within this reasoning, the COVID-19 drug label may be reserved for agents directed at host malfunctions specific for COVID-19. Overall, the misuse of terms complicates understanding of pandemic dynamics and mechanistic studies of the virus and the disease that inform actions on many levels, from personal to regional to global.

Although SARS-CoV-2 prompted the COVID-19 pandemic, it is humans who have 3 decisive roles that define its scale, dynamic, and impact; this consideration is applicable to any pandemic. Humans are a host for virus (SARS-CoV-2) reproduction (role one), which may trigger disease (COVID-19) development in the infected individual (role two). The (COVID-19 and SARS-CoV-2) pandemic tested resilience of the societal fabric that humans designed (role three). The genetics of SARS-CoV-2 and humans, as well as other biological factors (notably host age, prior infection or vaccination, and comorbidities), determined the outcomes of the virus reproduction and the host well-being [8,9]. By contrast, the societal outcomes were determined by local and global vulnerabilities to distress, besides direct adverse effects of the disease and virus transmission. For instance, limited availability of lifesaving oxygen balloons, ICU beds, and professional help was noted; however, these would be critical during other disruptive situations. Likewise, mitigation procedures such as emergency lockdowns were controversial measures that both saved and affected countless number of lives [10], reflecting painful trade-offs. Their implementation varied from very strict to relaxed among localities and countries, depending on many factors, including culture, geography, and understanding of COVID-19 and SARS-CoV-2.

Common reference to the societal disaster as COVID-19 during the ongoing pandemic is misleading, since the disease and disaster have different subjects, modalities, and dynamics. This may be particularly evident at the time of writing, when almost everyone has been exposed to SARS-CoV-2 and cases of COVID-19 have diminished but still exceed those of influenza. For vulnerable individuals, COVID-19 remains a threat. Yet, the pandemic may no longer be a global disaster since the disease burden is now accommodated without disrupting the fabric of human society, in contrast to the situation in 2020 to 2022. Communication and study of the societal disaster of this pandemic would be facilitated if it was recognized with a name separate from COVID-19, for instance, SARS-CoV-2-triggered disaster of *Homo sapiens*, or SADISA for short.

It is clear that the virus is not the disease and that neither is bound to trigger a disaster during a pandemic. We urge researchers, journalists, teachers, and policymakers to adopt the available terms that match the complexity of 3 main entities of the COVID-19 pandemic. This effort will improve communication about the pandemic and facilitate untangling the different roles of humans. During future pandemics, we suggest that a dedicated term for the societal disaster, encompassing all relevant effects irrespective of their direct link to the virus and the disease, be introduced from the disaster start. This entity-based concept is applicable to every virus, both during and between pandemics, and regardless of whether virus infections are associated with disease and social disaster. Its application will improve the connection of fundamental and clinical virology to studies of viromes and the virosphere.

## Abbreviations

AIDS: acquired immunodeficiency syndrome
COVID-19: coronavirus disease 2019
HIV-1: human immunodeficiency virus 1
MERS: Middle East respiratory syndrome
MERS-CoV: MERS coronavirus
SADISA: SARS-CoV-2-triggered disaster of *Homo sapiens*
SARS: severe acute respiratory syndrome
SARS-CoV: SARS coronavirus
SARS-CoV-2: severe acute respiratory syndrome coronavirus 2
